# Supplementary material for: Complexome profiling of the Chlamydomonas psb28 mutant reveals TEF5 as an early PSII assembly factor
Source: Plant Cell. 2025 Mar 17;37(6):koaf055. doi: 10.1093/plcell/koaf055 (PMC12164590; doi:10.1093/plcell/koaf055)
Supplement: koaf055_Supplementary_Data [file koaf055_supplementary_data.zip › psb28_interactive.zip!/plots/intensity/qualityPlots/peptides/SAR1_Cre11.g468300.t1.2.html]

### Cre11.g468300.t1.2

Trivial name: SAR1  
SeqCoverage [median]: 27.10  
Mass: 21.86 kDa  
  
 

| Sequence | MissedCleavage | Mass [Da] | wtIntensity | muIntensity | Score |
| --- | --- | --- | --- | --- | --- |
| AFDLGGHEIAR | 0 | 1184.59 | 2166.98 | 1693.26 | 87.57 |
| ILFLGLDNAGK | 0 | 1159.66 | 1259.82 | 0.00 | 118.50 |
| VNLSDQK | 0 | 802.42 | 0.00 | 621.61 | 104.45 |
| VVQHQPTQYPTSEEIQLGGINFK | 0 | 2612.32 | 0.00 | 2352.25 | 77.29 |
|  |  |  | 3426.8 | 4667.1 |  |
